# Detection of bovine leukemia virus, Epstein-Barr virus and human papillomavirus in breast cancer tissues of Egyptian patients

**DOI:** 10.1186/s13027-025-00674-y

**Published:** 2025-07-01

**Authors:** May Raouf, Salwa Kamal, Rawan Elsayed, Inass Zaki, Dina Kholeif

**Affiliations:** 1 Medical Microbiology and Immunology Department, Faculty of Medicine, Alexandria University, 0 Khartoum square, Azarita Medical campus, Alexandria, 21131 Egypt; 2 Pathology Department, Faculty of Medicine, Alexandria University, Alexandria, Egypt

**Keywords:** Breast cancer (BC), Bovine leukemia virus (BLV), Epstein-Barr virus (EBV), Human papillomaviruses (HPVs), HPV genotyping

## Abstract

**Background:**

Breast cancer (BC) remains one of the most common malignancies worldwide. Many viruses have been linked to BC; namely, Human papillomavirus (HPV), Epstein-Barr virus (EBV) and Bovine leukemia virus (BLV). However, a causal role is yet to be established.

**Objectives:**

To detect the prevalence of BLV, EBV and HPV sequences in BC tissue compared to BC-free tissue and correlate their presence with different pathological features of BC.

**Subjects and methods:**

: A retrospective case-control study was conducted on 75 FFPE (formalin fixed paraffin embedded) blocks of BC tissues and 25 of BC-free tissues obtained from Alexandria Main University Hospital pathology department archive. Demographic, medical, pathological data were retrieved from patients’ archival records. Hormonal receptor status, Real-time PCR for viral detection and HPV genotyping were done. Statistical analysis was done using SPSS software. The Chi-square test, Fisher’s Exact correction and Monte Carlo simulation were used for quantitative variables.

**Results:**

Invasive ductal carcinoma was the most predominant histologic type (85.3%). BLV, EBV and HPV were detected in (22.7% vs. 16%, 14.7% vs. 8%, 6.7% vs. 0%) BC vs. non-BC tissues respectively with HR HPV 16 detection. Lymphovascular invasion (LVI) and stage III were more commonly seen among tissues with positive viral detection vs. those which were negative (64.3% vs. 53% and 39% vs. 17% respectively). However, no single viral detection was found to be statistically significant in relation to clinicopathological parameters. Multiple viral co-existence was found in 18% of PCR positive cases which was significantly associated with younger age (*P* = 0.026).

**Conclusion:**

Low rate of viral presence was found in BC tissues. Nevertheless, LVI and stage III were more commonly seen in tissues with positive viral detection. Moreover, a synergetic relation between multiple viral existence and BC development in young age could be possible yet to be verified.

**Supplementary Information:**

The online version contains supplementary material available at 10.1186/s13027-025-00674-y.

## Introduction

Breast cancer (BC) is one of the most common malignancies in Egypt. It represents 32.04% of total cancers [1]. It is estimated that the number of new cases of BC worldwide would exceed 3 million by 2040, with low and middle-income countries seeing the biggest increase [2]. In comparison to BC cases in the west, North Africa and the Middle East has the highest incidence where women are affected at a relatively younger age (< 50 years) and usually present with advanced stage disease [3, 4]. The cancer burden is increasing due to the increased life expectancy of women, advanced detection methods, decreased maternal mortality, effective treatment of infectious diseases and certain environmental factors [5]. 

Risk factors for BC include demographics as female gender and advanced age, reproductive factors as early menarche, late menopause and nulliparity, hormonal factors as hormonal contraceptive pills as well as postmenopausal hormonal therapy, certain diet and lifestyle (alcohol consumption and cigarette smoking), family history and mutations of BC-related genes as *BRCA1* and *BRCA2* [6]. 

Although these risk factors are known, the direct cause of BC remains unknown. It has been proposed that certain infections could be considered as etiological factors of BC, highlighting the concept of oncobiome. However, to reach the conclusion of a direct viral cause of BC is very difficult. Many viruses have been linked to the development of BC; namely, bovine leukemia virus (BLV), Epstein-Barr virus (EBV), human papillomaviruses (HPVs), and mouse mammary tumor virus (MMTV) [7, 8]. 

It is estimated that 20% or more of human malignancies have an infectious cause with estimated higher percentages in developing countries with higher mortality rates and lower expected life span [9]. Oncogenesis can be achieved through multiple mechanisms such as direct effect of viral genes favoring the accumulation of mutations or preventing tumor cell removal by blocking the expression of apoptotic genes. Indirectly, viral infections cause reduction of the immune system defense mechanisms, immune evasion and chronic inflammation which lead to tumor formation [10]. 

HPV and EBV are both DNA viruses, while BLV is a delta retrovirus. These viruses have been linked to numerous malignancies such as cervical cancer, Burkitt’s lymphoma, nasopharyngeal carcinoma in humans and leukemia in cattle [11]. Bovine Leukemia virus, which is closely related to human T-lymphotropic virus type 1 (HTLV-1), causes bovine leukemia. In humans, it is an emerging zoonotic virus with potential transmission through intimate contact with infected animals and through infected dairy products and beef, yet to be verified [12]. It infects B-lymphocytes, T-lymphocytes and monocytes. BLV encodes an oncogene for the Tax protein, which activates nuclear factor kappa-light-chain-enhancer of activated B cells (NF-κB), prevents apoptosis and inhibits tumor suppressor genes resulting in accumulation of mutations due to unregulated cell proliferation with defective DNA damage repair. BLV potential oncogenic effect is suggested as there’s a higher prevalence of BC in countries with high milk consumption rates [13]. 

Epstein-Barr virus, also called human herpes virus 4 (HHV-4), mainly infects primary B-lymphocytes in childhood after contact with saliva. Epstein-Barr nuclear antigens (EBNA) and latent membrane protein 1 (LMP1) oncogenic proteins activates the HER2/HER3 pathways and NF-κB signaling that provoke cellular proliferation, inhibit apoptosis and induce angiogenesis, thus, leading to cancer development [14]. 

Human papillomavirus is a non-enveloped virus that infects epithelial cells and is primarily transmitted through sexual activity. It is classified into high-risk types (e.g. 16, 18, 31, 33, 35, 39, 45, 51, 59) and low-risk types (e.g. 6,11) according to its oncogenic potential. HPV E6 and E7 viral oncoproteins cause inactivation of P53 and Rb tumor suppressor genes respectively [15]. 

Complex biological interactions promoting oncogenesis due to multiple viral coexistence are hypothesized for BC. BLV, EBV and HR HPV viruses have substantial evidence, but not conclusive, in the development of breast cancer according to expanded Hill causal criteria [11]. The 3 viruses were detected in benign breast tissues about a decade before the development of BC in the same patients [16]. The synergistic effect between HR HPV and EBV was widely investigated in epithelial tumors. It is hypothesized that the presence of EBV and HPV together can increase the oncogenic potential of HPV. This EBV influence is due to the production of an IL-10 homolog called BCRF1 which modulates the immune response against HPV [11]. BLV TAX, EBV LMP1 and HPV E6 and E7 enhance unregulated cell proliferation [8]. The 3 viruses stimulate NF-κB enhancer of activated B cells leading to inhibition of immune surveillance system [10, 17]. Moreover, EBV and HPV are known to alter the antiviral enzyme apolipoprotein B messenger RNA editing enzyme, catalytic polypeptide-like (APOBEC3) inducing cellular mutagenesis [7, 8]. Also, Chronic inflammation is a common feature of the 3 viruses due to oxidative stress which aids in cell transformation and accumulation of mutations [10]. 

Detection and identification of these three viruses in BC is crucial to detect any correlation. These methods include amplification techniques such as different types of polymerase chain reaction (PCR), in situ hybridization, immunohistochemistry and genome sequencing. However, PCR remains the most common method used for viral detection [11]. 

The aim of this study was to detect BLV, EBV and HPV nucleic acids among tissues of Egyptian BC patients, and to correlate the presence of each virus to the clinicopathological parameters of the disease. To our best knowledge, this is the first study exploring BLV, EBV and HPV together in breast cancer tissues worldwide.

## Patients and methods

### Study setting

A retrospective case control study conducted on 75 BC Formalin-fixed, paraffin-embedded (FFPE) tissue blocks with proven histopathological evidence and complete data sheets were included as well as 25 BC free FFPE blocks (6 accessory breast, 3 reduction mammoplasty tissues, 11 fibroadenoma, 5 benign cystic lesions) that were obtained after surgical procedures at Alexandria Main University Hospital and archived in pathology department during the period from April 2022 to April 2023. Premalignant breast tissues, safety margins and BC tissues with incomplete data sheets were excluded.

The sample size was calculated using the G*Power software (version 3.1.9.7) with 80% power and 95% confidence level [18]. 

### Data collection from patients’ archival pathology records

Demographic data (age, marital status, age of menarche and menopause), medical history of patients (Diabetes, cardiac diseases and hypertension) and family history of BC were retrieved. Surgical data as type of procedure done, presurgical treatment and response were also collected. Data from pathology archive reports according to the CAP protocol for breast carcinoma (according to WHO classification of breast carcinoma) [19], histologic grading according to Nottingham Histologic Score (the Elston-Ellis modification of Scarff-Bloom-Richardson grading system) [20] were obtained.

### BC hormone receptor status and quantification: [21]

Hormone receptors; estrogen and progesterone (ER, PR) and Her2-neu were detected by immunohistochemistry, their expression was visualized using the streptavidin-biotin-immunoenzymatic antigen detection system which was performed according to manufacturer’s protocol. The detection system was provided by Lab Vision Corporation (ThermoFisher, Fremont, USA). For ER and PR immunostaining, normal ductal epithelial cells served as internal positive control. While for Her2-neu immunostaining, normal ductal epithelial cells served as internal negative control.

### Molecular study protocol

#### Genomic DNA extraction

Extraction and purification of genomic DNA from FFPE tissues was done using QIAamp^®^ DNA FFPE Tissue (QIAGEN, Hilden, Germany) according to the manufacturer ‘s instructions. The quantity and purity of the extracted genomic DNA was evaluated using The NanoDrop^®^ 1000 Spectrophotometer (ThermoFisher Scientific, Massachusetts, USA) and stored at -20◦c.

## DNA amplification

The human housekeeping β-globin gene was used as an internal control for DNA integrity. Specific PCR primers for each of the viruses were provided from Invitrogen™ by ThermoFisher Scientific Inc. (ThermoFisher, Fremont, USA) as follows. They were examined for specificity by searching through databases of viral genomes using BLAST (Basic Local Alignment Search Tool) [22]. 

Human housekeeping β-globin gene (110 bp).

PCO3 5’CTTCTGACACAACTGTGTTCACTAGC3’.

PCO4 5’TCACCACAACTTCATCCACGTTCACC3’.

BLV tax oncoprotein encoding gene (114 bp) [13], 

tax F 5’ATGTCACCATCGATGCCTGG3’.

tax R 5’CATCGGCGGTCCAGTTGATA3’.

EBV (HHV4) Epstein-Barr nuclear antigen encoding gene (310 bp) [23].

EBNA F5’CATCGCAGGGTTCTTACCAT3’.

EBNA R 5’GAAGAAACAGCCTCCTGCAC3’.

HPV L1 protein encoding gene was used for wide range of HPV types (150 bp) [24].

GP5 + 5’TTTGTTACTGTTGTTGATACTAC3’.

GP6 + 5’GAAAAATAAACTGTAAATCATATTC3’.

The total reaction volume was 20 µl with 10 µl of master mix, 1 µl of each of the forward and reverse primers (10 Picomoles), 2 µl of template DNA and 6 µl of DNase-Free Distilled Water. Amplification was done using “2xSensiFAST SYBR^®^ No-ROX mix, (Meridian Bioscience Inc., Ohio, USA) according to manufacturer’s instructions [25]. It is optimized as hot-start polymerase included in the kit minimizes non-specific amplification, and also by calculating annealing temperature through primer blasting.” [22].

Positive controls included BLV first positive PCR product that was confirmed by running the PCR product on electrophoresis 2% agar. For EBV and HPV: Lab proven EBV associated nasopharyngeal carcinoma and Lab proven HPV associated cervical cancer obtained from the pathology department archive were used as a positive control respectively.

The following PCR conditions were followed: polymerase activation was established at 95 °C for 2 min, followed by denaturation (40 cycles at 95 °C for 5 s), followed by for 40 cycles of annealing at 55**°**C for BLV and Human β-globin, 58 °C for EBV and 45 °C for HPV, finally an extension step was carried out at 72 °C for 20 s. The four PCR reactions were done separately using the Rotor-Gene™ Q (Qiagen, Hilden, Germany) real-time PCR machine.

Verification of the amplification product was done through analysis of the melt profile and comparing the amplification plot to positive and negative controls curves. Also, Amplification was verified by running the PCR products on 2% agarose gel.

### HPV genotyping[26]

Genotyping was done for all 5 HPV positive samples using the multiplex real-time PCR assay Allplex™ HPV28 Detection (Seegene, Inc., Seoul, South Korea). DNA concentration ≥ 50 ng/ul and adequate volume (≥ 5 ul) were required for genotyping.

### Statistical analysis of the data [27]

Data was fed to the computer and analyzed using IBM SPSS software package version 20.0. (Armonk, NY: IBM Corp). Qualitative data were described using numbers and percentages. The tests used were the Chi-square test for categorical variables, to compare between different groups, Fisher’s Exact correction for small size samples (more than 20% of the cells have expected count less than 5). Monte Carlo simulation was used as a computational method for normalization and uncertainty analysis. The significance of obtained results was judged at the 5% level. Univariate regression test values of *P* < 0.05 were followed by multivariate analysis.

## Results

### Patients’ characteristics, medical and family history findings

Demographic data were collected from patients’ archival pathology records. About two thirds of BC cases were 50 years and older versus half of BC free cases (62.7%, *n* = 47 vs. 52%, *n* = 13; Table 1).

**Table 1:** Demographic characteristics of breast cancer cases and controls with univariate and multivariate regression analysis (*n*=100)

	BC Cases (*n* = 75)		BC free cases (*n* = 25)		χ^2^	*P*	Univariate		Multivariate #	
	No.	%	No.	%			*P*	OR (95% C.I)	*p*	OR (95% C.I)
**Age (years)**								1.034 (0.992 – 1.077)		
<50	28	37.3	13	52.0	1.667	0.197	0.117			
≥50	47	62.7	12	48.0						
**Menopause**								17.912 (5.405 – 59.357)	0.113	3.641 (0.735 – 18.038)
Pre	17	22.7	21	84.0	29.938^*^	<0.001*	0.001*			
Post	58	77.3	4	16.0						
**Age of menarche (years)**					0.882	^MC^p= 0.579	0.937	0.981 (0.604 – 1.592)		
≤12	22	29.3	5	20.0						
>12	53	70.7	20	80.0						
**Medical history**										
Free	40	53.3	21	84.0	7.412^*^	0.006*	0.010*	0.218 (0.068 – 0.695)	0.814	1.358 (0.106 – 17.444)
Hypertension	28	37.3	2	8.0	7.683^*^	0.006*	0.013*	6.851 (1.500 – 31.286)	0.785	1.527 (0.073 – 31.972)
Cardiac	9	12.0	1	4.0	1.333	FEp=0.444	0.273	3.273 (0.394 – 27.215)		
Diabetes	13	17.3	1	4.0	2.769	FEp=0.179	0.129	5.032 (0.624 – 40.597)		
**Family history**						FEp= 0.227	0.150	3.119 (0.664 – 14.648)		
Negative	59	78.7	23	92.0	2.258					
Positive	16	21.3	2	8.0						

BC: breast cancer, χ^2^: Chi square test, FE: Fisher Exact, MC: Monte Carlo

OR: Odd`s ratio, C.I: Confidence interval

P: P value for comparing the two studied groups, *: Statistically significant at *p* ≤ 0.05

#: All variables with *p*<0.05 were included in the multivariate

All specimens were from married females (currently or previously). The mean age of menarche was the same in both groups (12.92 ± 1 year). About three quarters of BC cases were menopausal versus one quarter of controls which was statistically significant (*P* **<** 0.001).

More than half of BC cases (53.3%, *n* = 40) versus 84% (n=) of BC free cases had free medical history with statistically significant difference (*P* = 0.006). Hypertension was reported among 37.3% (*n* = 28) of BC cases versus 8% (*n* = 2) in BC free cases with statistically significant difference (*P* = 0.006). About 21.3% of BC cases (*n* = 16) reported positive family history of BC versus 8% of BC free cases (*n* = 2). Univariate analysis was done for the previous parameters and statistically significant difference was detected between cases versus controls regarding post-menopausal state, being free of medical conditions or having hypertension (OR: 17.912, 95% CI: 5.405–59.357, *P* = 0.001), (OR:0.218, 95% CI:0.068 − 0.695, *P* = 0.010), (OR:6.851, 95% CI:1.500–31.286, *P* = 0.013) respectively. in the multivariate analysis, the previous statistically significant values were not retained (OR: 3.614, 95% CI: 0.735–18.038, *P* = 0.113), (OR:1.358, 95% CI:0.106 − 17.444, *P* = 0.814), (OR:1.527, 95% CI:0.073–31.972, *P* = 0.785).

### Hormonal receptor status among the studied BC cases

**Fig. 1:**
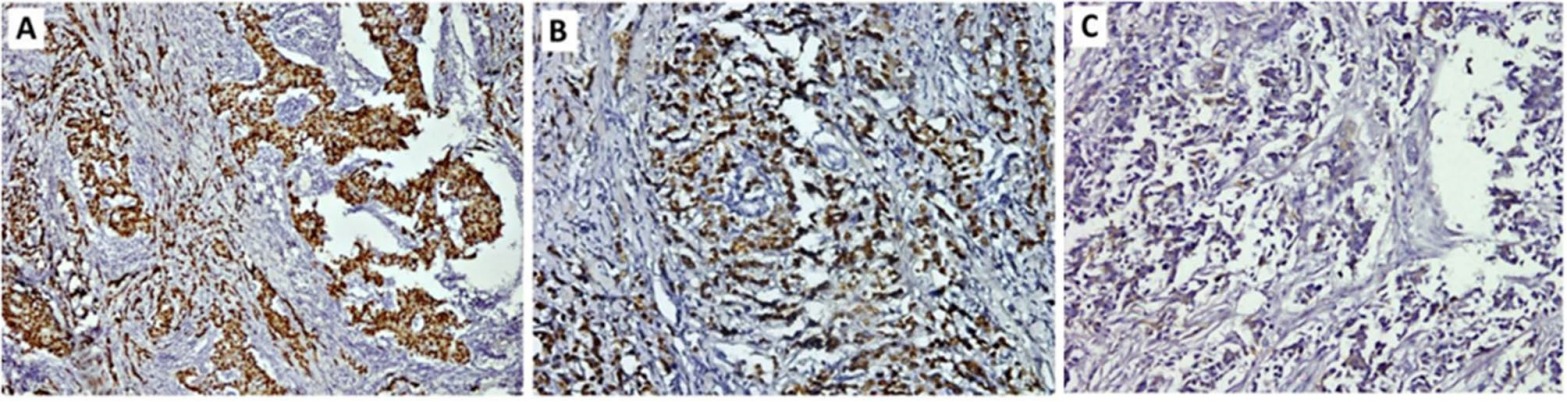
A case of invasive ductal carcinoma of no special type NST, G2 showing **A**: positive nuclear staining for ER antibody, **B**: positive nuclear staining for PR antibody, **C**: negative membranous staining (score 0) for Her2-neu antibody (Immunoperoxidase A: x100, B: x200, C: x200)

**Fig. 2:**
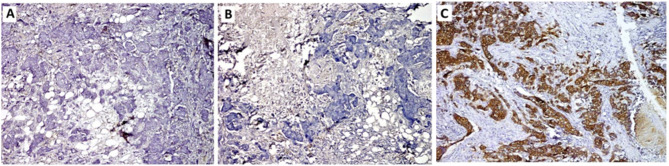
A case of Invasive ductal carcinoma of no special type (NST), G3, showing **A**: negative nuclear staining for ER antibody, **B**: negative nuclear staining for PR antibody, **C**: positive membranous staining (score 3+) for Her2-neu antibody (Immunoperoxidase x100)

More than half of BC tissues were ER + PR positive and HER2 neu negative (57.3%, *n* = 43; Table 2) whereas only 8% (*n* = 6) were HER2 neu positive and one quarter (24%, *n* = 18) were triple negative. (Figures 1A-C and 2A-C).

**Table 2:** Hormonal receptor status among the studied breast cancer cases (*n* = 75)

Hormonal receptor	No.	%
**ER only**	5	6.7
**PR only**	0	0.0
**HER2-neu only**	6	8.0
**ER+PR positive+HER2-neu negative**	43	57.3
**ER + HER2-neu**	2	2.7
**ER + PR + HER2-neu**	1	1.3
**Triple negative**	18	24.0

ER: estrogen receptor, PR: progesterone receptor, HER2-neu: human epidermal growth factor receptor

### Pathological assessment of BC tissues

Modified radical mastectomy (MRM) was done to 93.3% (*n* = 70) of the studied BC cases. The most predominant histologic type assessed was invasive ductal carcinoma (IDC) of no special type (NST) (85.3%, *n* = 6; Table 3). Most of the assessed tumors were between 2 and 5 cm (80%, *n* = 60). Grade 2 was the most common tumor grade (77.3%, *n* = 58). Ductal carcinoma in situ (DCIS) was noted in 42.7% (*n* = 32) of tumors. More than half of the tumors showed Lymphovascular invasion (57.3%, *n* = 43).

**Table 3:** Different histopathological features in breast cancer cases (*n* = 75)

Histopathology	No.	%
**Tumor Size**		
<2 cm 2–5 cm >5 cm	12 60 3	16.0 80.0 4.0
**Histologic Type**		
IDC	64	85.3
ILC	5	6.7
IMC	6	8.0
**Tumor Grade**		
1	3	4.0
2	58	77.3
3	14	18.7
**DCIS**		
Absent	43	57.3
Present	32	42.7
**Lymphovascular invasion**		
Negative	32	42.7
Positive	43	57.3
**Distant metastasis**		
Negative	75	100.0
Positive	0	0.0
**pTNM Staging**		
I	21	28.0
II	35	46.7
III	19	25.3

IDC: Invasive ductal carcinoma, ILC: Invasive lobular carcinoma, IMC: Invasive mammary carcinoma, DCIS: Ductal carcinoma insitu, pTNM: Pathological Tumor-Node-Metastasis

**Fig. 3:**
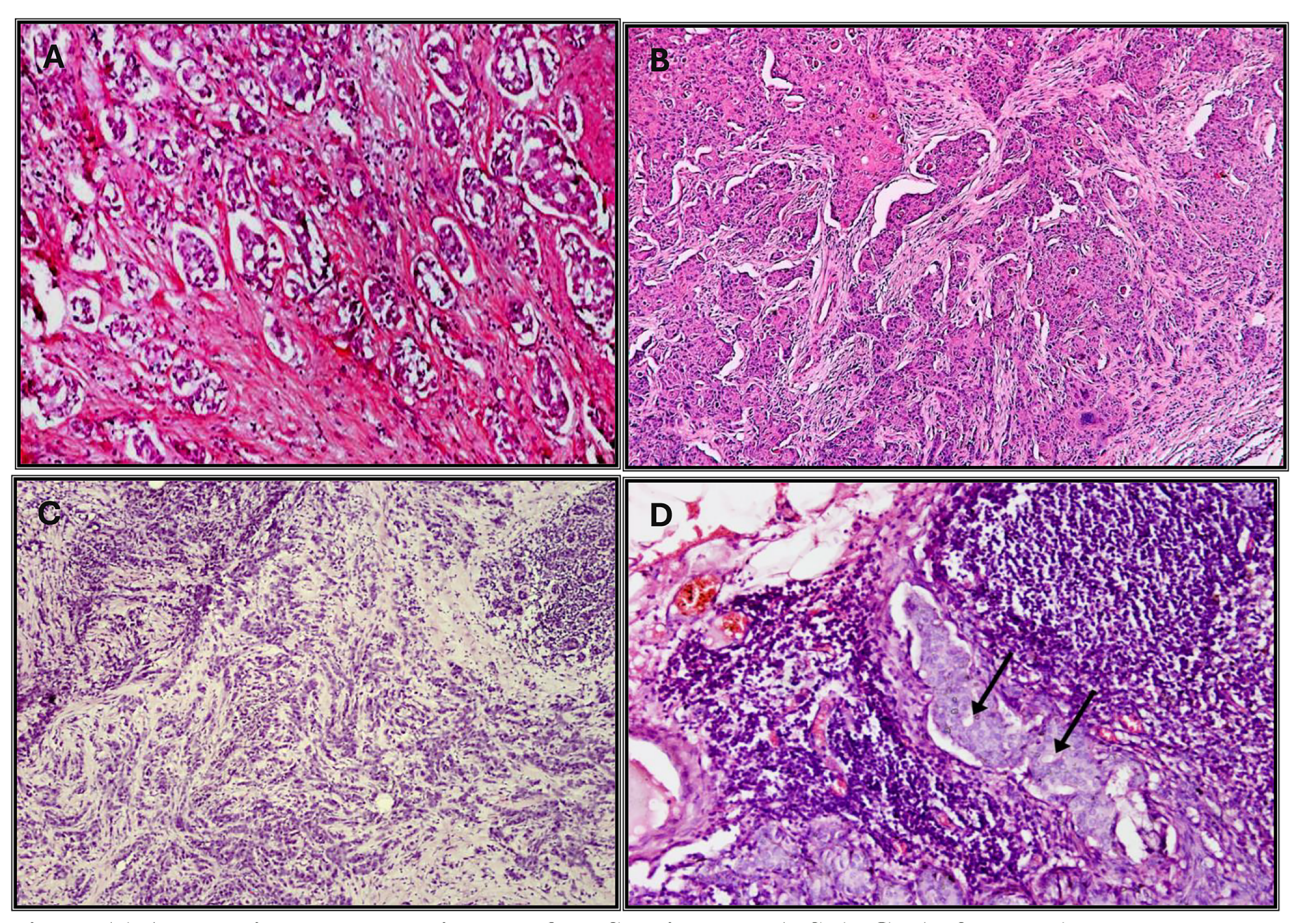
**A**: Invasive ductal carcinoma of No Special Type (NST), G2 (H&E x100), **B**: Invasive ductal carcinoma of No Special Type (NST), G3 (H&Ex100), **C**: Invasive lobular carcinoma (H&Ex100), **D**: Lymph node invasion. Note the microacinar pattern (arrows) (H&Ex200)

Regarding the pTNM staging of the tumor, stage II constituted about half (46.7%, *n* = 35) of the studied BC cases. None of the tumors were metastatic. (Fig. 3A-D).

### BLV, EBV and HPV viral DNA detection by real time PCR results

Real-time PCR was performed on DNA extracted from 75 BC tissue and 25 BC free tissues and followed by analysis of the melt profile (melting curve analysis, Tm) (supplementary 1) and detection of amplification products bands by 2% gel electrophoresis (supplementary 2).

Regarding amplification of the human housekeeping β-globin gene, all samples (100%, *n* = 100) were positive denoting satisfactory DNA extraction.

**Fig. 4:**
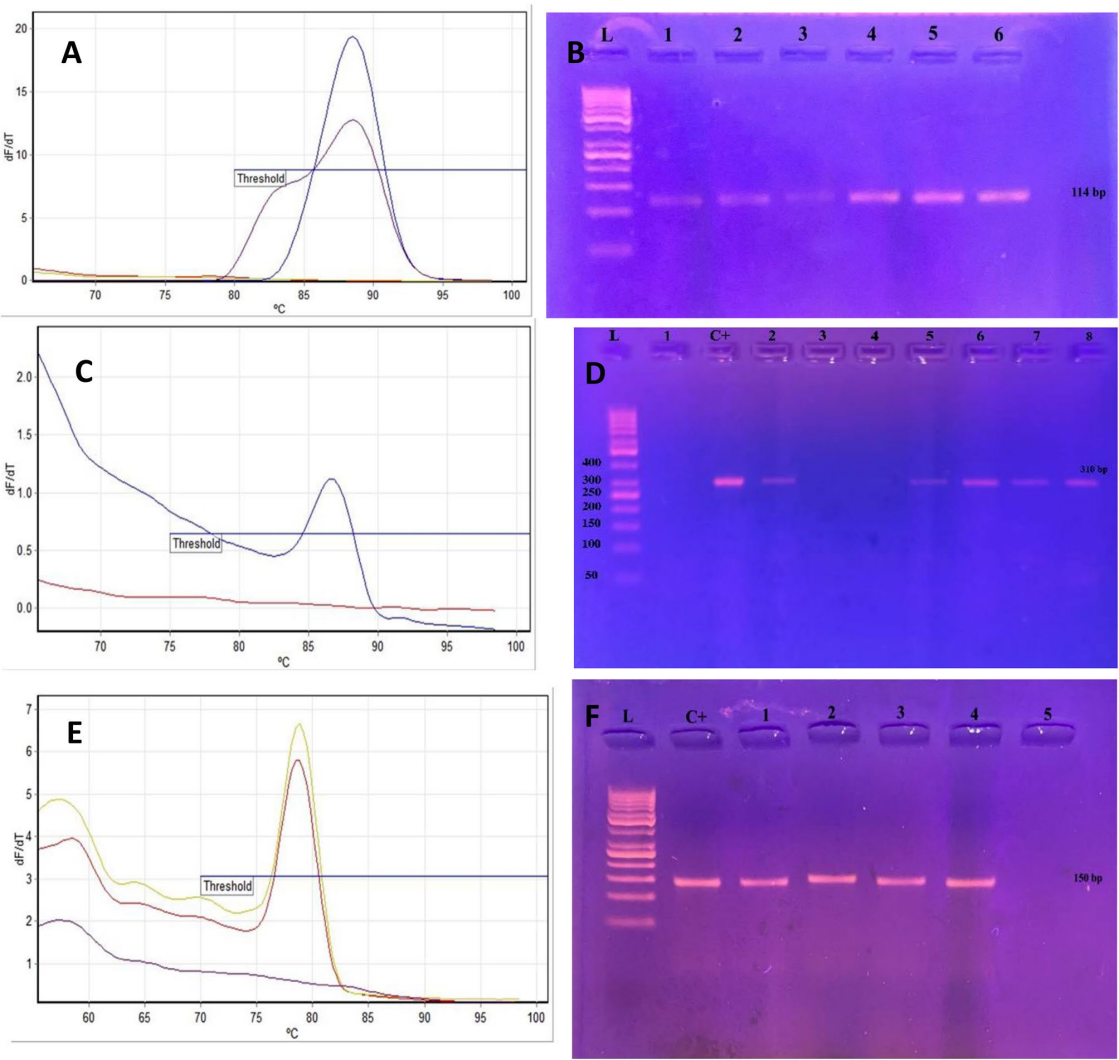
**A**: Positive and negative samples for BLV TAX gene by real-time PCR (Tm 88 ± 0.5 °C), **B**: positive samples for BLV TAX gene (114-bp PCR product) on 2% agarose gel. (L: 50 bp gel ladder. 1–6: positive samples.) **C**: Positive and negative samples for EBV EBNA gene by real-time PCR (Tm 86 ± 0.5 °C), **D**: Positive samples for EBV EBNA gene (310-bp PCR product) on 2% agarose gel. (L: 50-bp DNA ladder, C+: positive control, 2, 5–8: positive samples, 1, 3–4: Negative samples). **E**: Positive and negative samples for HPV L1 gene by real-time PCR (Tm 79 ± 0.5 °C), **F**: Positive samples for HPV L1 gene (150-bp PCR product) on 2% agarose gel. (L: 50-bp DNA ladder, C+: positive control, 1–4: positive samples, 5: Negative sample)

BLV *Tax* gene was detected in 22.7% (*n* = 17) of BC tissues versus 16% (*n* = 4) in BC free tissues (Table 4) with 114-bp PCR product band detected by 2% agarose gel electrophoresis. (Fig. 4A-B).

**Table 4:** Detection of bovine leukemia virus, Epstein-Barr virus and human papillomavirus among breast cancer versus breast cancer free tissues with univariate regression analysis

	Total (*n* = 100)		BC (*n* = 75)		BC free (*n* = 25)		χ^2^	*p*	Univariate regression	
	No.	%	No.	%	No.	%			*p*	OR (95% C.I)
**BLV**	21	21.0	17	22.7	4	16.0	0.502	0.478	0.481	1.539 (0.464 – 5.099)
**EBV**	13	13.0	11	14.7	2	8.0	0.737	^FE^*p*=0.508	0.398	1.977 (0.407 – 9.598)
**HPV**	5	5.0	5	6.7	0	0.0	1.754	^FE^*p*=0.327	^-^	^---^

BC: breast cancer, BLV: Bovine leukemia virus, EBV: Epstein Barr virus, HPV: Human papillomavirus

χ2: Chi square test, FE: Fisher Exact, P: p value, OR: Odd`s ratio, C.I: Confidence interval

As regards EBV *EBNA* gene, it was detected in 14.7% (*n* = 11) of BC tissues versus 8% (*n* = 2) in control tissues with 310-bp PCR product band detection (Fig. 4C-D).

HPV *L1* gene was present in 6.7% (*n* = 5) of BC tissues with 150-bp PCR product band detection. HPV DNA was not detected in BC free tissues (Fig. 4E-F).

Viral DNA was not detected in 62.7% (*n* = 47) of BC and 76% (*n* = 19) of BC free tissues. The detection rate of viruses showed no statistically significant difference between tumorous and malignant free tissues for BLV (22.7% vs. 16%, *P* = 0.478), EBV (14.7% vs. 8%, *P* = 0.508) and HPV (6.7% vs. 0%, *P* = 0.327). Univariate analysis was done for the viral prevalences detected with no significant difference found.

### HPV genotyping results

**Fig. 5:**
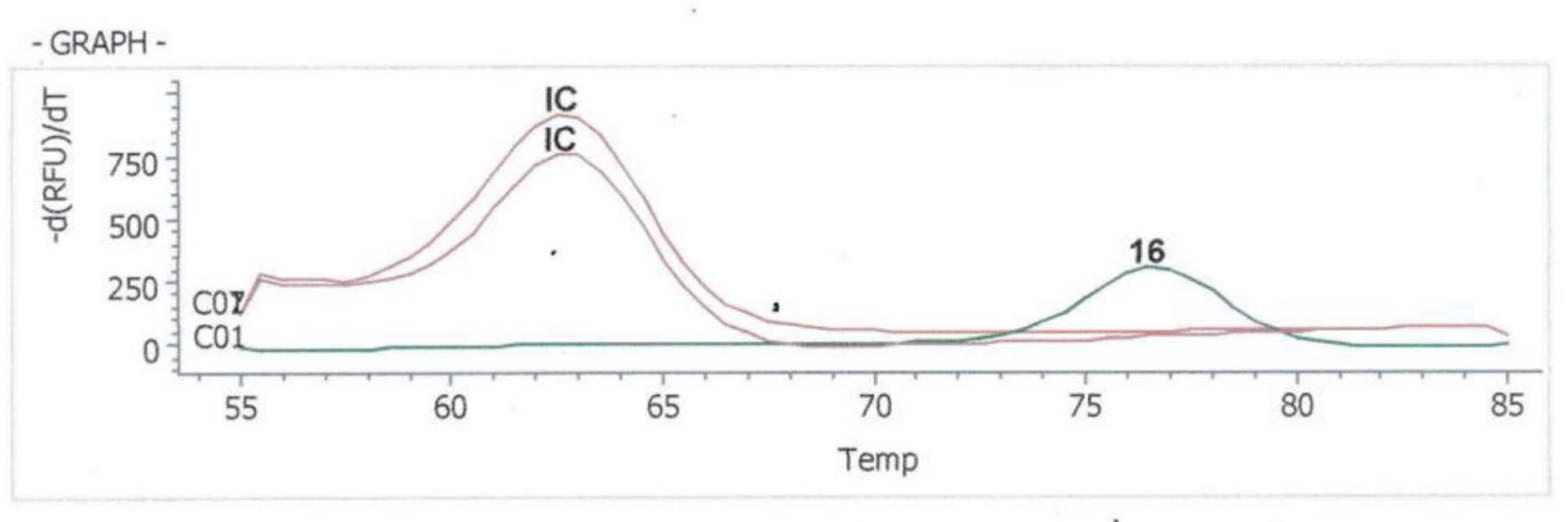
HPV 16 positive sample by genotyping. (16: Positive sample for genotype HPV 16, IC: Internal Control)

HPV genotyping was done for the 5 HPV positive samples. Only two samples having high DNA concentrations (108 and 50.2 ng/ul) and adequate volumes, as per test requirement, were positive for the high-risk HPV 16. (Fig. 5). The other 3 cases showed negative results due to low DNA concentrations (43.6, 40.9 and 23.1 ng/ul) and small volumes (supplementary 1).

### Association between BLV, EBV and HPV detection and pathological data

BLV, EBV and HPV detection were assessed in relation to different pathological features (Table 5).

**Table 5:** Association between bovine leukemia virus, epstein barr virus and human papillomavirus with different pathological features in breast cancer tissues (*n* = 75)

	BLV			EBV			HPV		
	Positive (*n* = 17)	Negative (*n* = 58)	*p*	Positive (*n* = 11)	Negative (*n* = 64)	*p*	Positive (*n* = 5)	Negative (*n* = 70)	*p*
**Age (years)**									
<50	6 (35.3%)	22 (37.9%)	^χ2^p= 0.843	6 (54.5%)	22 (34.4%)	^χ2FE^p= 0.311	1 (20.0%)	27 (38.6%)	^χ2FE^p= 0.645
≥50	11 (64.7%)	36 (62.1%)		5 (45.5%)	42 (65.6%)		4 (80.0%)	43 (61.4%)	
**ER Hormonal Status**									
Negative	5 (29.4%)	19 (32.8%)	^χ2^p= 0.795	5 (45.5%)	19 (29.7%)	^χ2FE^p= 0.314	1 (20.0%)	23 (32.9%)	^χ2FE^p= 1.000
Positive	12 (70.6%)	39 (67.2%)		6 (54.5%)	45 (70.3%)		4 (80.0%)	47 (67.1%)	
**PR Hormonal Status**									
Negative	8 (47.1%)	23 (39.7%)	^χ2^p= 0.586	6 (54.5%)	25 (39.1%)	^χ2FE^p= 0.509	1 (20.0%)	30 (42.9%)	^χ2FE^p= 0.397
Positive	9 (52.9%)	35 (60.3%)		5 (45.5%)	39 (60.9%)		4 (80.0%)	40 (57.1%)	
**HER2-neu Hormonal Status**									
Negative	13 (76.5%)	53 (91.4%)	^χ2FE^p= 0.196	8 (72.7%)	58 (90.6%)	^χ2FE^p= 0.121	5 (100.0%)	61 (87.1%)	^χ2FE^p= 1.000
Positive	4 (23.5%)	5 (8.6%)		3 (27.3%)	6 (9.4%)		0 (0.0%)	9 (12.9%)	
**Triple negative**									
No	14 (82.4%)	43 (74.1%)	^χ2FE^p= 0.748	8 (72.7%)	49 (76.6%)	^χ2FE^p= 0.719	4 (80.0%)	53 (75.7%)	^χ2FE^p= 1.000
Yes	3 (17.6%)	15 (25.9%)		3 (27.3%)	15 (23.4%)		1 (20.0%)	17 (24.3%)	
**Histologic Type**									
IDC	14 (82.4%)	50 (86.2%)	^χ2MC^p= 0.153	9 (81.8%)	55 (85.9%)	^χ2MC^p= 0.818	5 (100.0%)	59 (84.3%)	^χ2MC^p= 1.000
ILC	0 (0.0%)	5 (8.6%)		1 (9.1%)	4 (6.3%)		0 (0.0%)	5 (7.1%)	
IMC	3 (17.6%)	3 (5.2%)		1 (9.1%)	5 (7.8%)		0 (0.0%)	6 (8.6%)	
**Tumor Size**									
<2 cm	1 (5.9%)	11 (19.0%)	^χ2MC^p= 0.115	1 (9.1%)	11 (17.2%)	^χ2MC^p= 0.503	0 (0.0%)	12 (17.1%)	^χ2MC^p= 0.662
2–5 cm	14 (82.4%)	46 (79.3%)		9 (81.8%)	51 (79.7%)		5 (100.0%)	55 (78.6%)	
>5 cm	2 (11.8%)	1 (1.7%)		1 (9.1%)	2 (3.1%)		0 (0.0%)	3 (4.3%)	
**Histologic Grade**									
1	0 (0.0%)	3 (5.2%)	^χ2MC^p= 0.383	0 (0.0%)	3 (4.7%)	^χ2MC^p= 0.655	0 (0.0%)	3 (4.3%)	^χ2MC^p= 1.000
2	12 (70.6%)	46 (79.3%)		8 (72.7%)	50 (78.1%)		4 (80.0%)	54 (77.1%)	
3	5 (29.4%)	9 (15.5%)		3 (27.3%)	11 (17.2%)		1 (20.0%)	13 (18.6%)	
**Lymphovascular Invasion**									
Negative	5 (29.4%)	27 (46.6%)	^χ2^p= 0.209	5 (45.5%)	27 (42.2%)	^χ2FE^p= 1.000	1 (20.0%)	31 (44.3%)	^χ2FE^p= 0.386
Positive	12 (70.6%)	31 (53.4%)		6 (54.5%)	37 (57.8%)		4 (80.0%)	39 (55.7%)	
**pTNM Staging**									
I	4 (23.5%)	17 (29.3%)	^χ2MC^p= 0.635	3 (27.30%)	18 (28.1%)	^χ2MC^p= 0.642	1 (20.0%)	20 (28.6%)	^χ2MC^p= 0.151
II	7 (41.2%)	28 (48.3%)		4 (36.4%)	31 (48.4%)		1 (20.0%)	34 (48.6%)	
III	6 (35.3%)	13 (22.4%)		4 (36.4%)	15 (23.4%)		3 (60.0%)	16 (22.9%)	

χ2: Chi square test, MC: Monte Carlo, FE: Fisher Exact

*: Statistically significant at *p* ≤ 0.05

IDC: Invasive ductal carcinoma, ILC: Invasive lobular carcinoma, IMC: Invasive mammary carcinoma, ER: estrogen receptor, PR: progesterone receptor, HER2-neu: human epidermal growth factor receptor, pTNM: Pathological Tumor-Node-Metastasis

BLV and HPV positive specimens were more detected in females above 50 years (64.7% and 80%) respectively while EBV was more detected with younger age (54.5%).

Grade 3 was more noted in BLV and EBV positive BC specimens versus negative BC specimens (29.4% vs. 15.5% and 27.3% versus 17.2%) respectively.

LVI was more commonly seen among BC cases positive for viral detection (64.3% vs. 53.2% in BC negative for viral detection). A relation between viral detection and late stage III was noted (39.3% vs. 17% with negative viral detection) especially with HPV positivity (60% vs. 22.9%). Although there were notable differences, no single viral detection was found to show statistically significant difference in relation to clinicopathological parameters.

### Co-detection of multiple viruses’ assessment

The co-detection of multiple viruses was assessed in Table 6. A total of 28 BC cases (37.3%) showed positive viral PCR results. Of which, 23cases (82%) were positive for only a single virus while 5 BC (18%) cases were positive for two viruses simultaneously. BLV and EBV were detected simultaneously in 4 cases while one BC case was positive for both EBV and HPV. The three studied viruses were not detected simultaneously. Viral codetection was not detected in BC free tissues.

**Table 6:** A comparison between single vs. multiple viral co-existence and its relation to different pathological features in breast cancer patients

	Co-existence		Test of Sig.	*p*
	Single virus (*n* = 23)	Multiple virus (*n* = 5)		
**Age (years)**				
<50	5 (21.7%)	4 (80.0%)	χ^2^= 6.392^*^	^FE^p= 0.026^*^
≥50	18 (78.3%)	1 (20.0%)		
**ER**				
Negative	7 (30.4%)	2 (40.0%)	χ^2^= 0.172	^FE^p= 1.000
Positive	16 (69.6%)	3 (60.0%)		
**PR**				
Negative	9 (39.1%)	3 (60.0%)	χ^2^= 0.730	^FE^p= 0.624
Positive	14 (60.9%)	2 (40.0%)		
**HER2-neu**				
Negative	20 (87.0%)	3 (60.0%)	χ^2^= 2.035	^FE^p= 0.207
Positive	3 (13.0%)	2 (40.0%)		
**Triple negative**				
No	18 (78.3%)	4 (80.0%)	χ^2^= 0.007	^FE^p= 1.000
Yes	5 (21.7%)	1 (20.0%)		
**Histologic Type**				
IDC	20 (87.0%)	4 (80.0%)	χ^2^= 1.471	^MC^p= 0.564
ILC	1(4.3%)	0 (0.0%)		
IMC	2 (8.7%)	1 (20.0%)		
**Tumor Size**				
<2 cm	2 (8.7%)	0 (0.0%)	χ^2^= 1.994	^MC^p= 0.570
2–5 cm	20 (87.0%)	4 (80.0%)		
>5 cm	1 (4.3%)	1 (20.0%)		
**Histologic Grade**				
I	0 (0.0%)	0 (0.0%)	χ^2^= 0.730	^FE^p= 0.574
II	18 (78.3%)	3 (60.0%)		
III	5 (21.7%)	2 (40.0%)		
**Lymphovascular invasion**				
Negative	9 (39.1%)	1 (20.0%)	χ^2^= 0.655	^FE^p= 0.626
Positive	14 (60.9%)	4 (80.0%)		
**pTNM Staging**				
I	6 (26.1%)	1 (20.0%)	χ^2^= 0.320	^MC^p= 1.000
II	8 (34.8%)	2 (40.0%)		
III	9 (39.1%)	2 (40.0%)		

χ2: Chi square test, MC: Monte Carlo, FE: Fisher Exact

*: Statistically significant at *p* ≤ 0.05

IDC: Invasive ductal carcinoma, ILC: Invasive lobular carcinoma, IMC: Invasive mammary carcinoma, ER: estrogen receptor, PR: progesterone receptor, HER2-neu: human epidermal growth factor receptor, pTNM: Pathological Tumor-No

A comparison between BC cases positive for a single virus versus those positive for multiple viruses showed that the existence of a single virus is more common in the older age group (≥ 50 years), while multiple viral co-detection was more common in the younger age group (< 50 years) with a statistically significance difference (*P* = 0.026). A higher grade (40% vs. 21.7%) was detected with multiple viruses but with no statistically significance difference.

## Discussion

An infectious causative factor for BC has not been established, yet the search for direct viral causes has been the target of many studies in the past few years [7, 11, 28, 29]. 

Proving evidence for a viral cause for BC would be a breakthrough in the fields of microbiology and oncology as it will help to better understand the mechanism of disease development, identify biomarkers to screen high risk patients, develop prognostic indicators, prevent the disease through vaccination or elimination of these viruses and can provide new adjuvant antiviral treatment modalities [30]. Among those which stand out in BC are BLV, EBV and HPV which were our study focus.

Regarding the current study’s demographic data, among 75 BC patients, the highest age group in prevalence was fifty years old with mean age of 53.76. This is nearly matching the mean age two meta-analyses conducted in Africa, in Egypt by Azim et al., and a study in Saudi Arabia (50.2, 50.4, and 55.68 years respectively) [4, 31, 32]. However, it is lower than developed countries. This could be due to higher mortality rates as more advanced tumors are seen with expected shorter life spans [5]. 

Postmenopausal state was more common in BC cases vs. controls with detected significant statistical difference (*P* **=** 0.006). Regarding medical history, hypertension was the most prevalent associated disease and was reported in 37.3% of BC patients vs. 8% in controls with statistically significant result (*P* **=** 0.006). Hypertension could be associated with the development of BC and other types of cancers as reported in a meta-analysis by *Han et al.* and in study by *Fan et al.* especially in postmenopausal females [33, 34]. This association could be due to multiple factors, including common pathophysiological mechanisms including dysfunction of the adipose tissue of the breast as a result of enlargement of fat cell size and infiltration of fat cells by macrophages leading to chronic inflammation which is a predisposition for BC development [35]. Moreover, postmenopausal obesity increase estrogen level, which is a risk factor for developing BC. Another possible mechanism is that hypertension can impact the control of cell turnover, leading to inhibition of apoptosis and the promotion of BC [33]. 

As regards family history, 21.3% of the studied BC patients had positive family history versus 8% of controls. Globally, approximately 30 to 40% of BC cases have a positive family history of BC [36]. 

Hormonal receptor status was done to all the studied BC cases. More than half of the studied BC tissues (57.3%) were ER + PR positive and HER2-neu negative. This matches an Egyptian study by *khader et al.* [37]. Triple negative BC (TNBC) comprised 24% of the studied BC tissues. Worldwide, TNBC represents up to 20% of all BC subtypes. Compared to other molecular subtypes, TNBC frequently is the most aggressive subtype, with greater rates of recurrence and chances of metastasis [38]. 

Drifting to the pathological parameters, 85.3% were IDC while 6.7% were ILC. This runs in parallel with the meta-analysis conducted by *Azim et al.* in which IDC and ILC represented 87% and 7% respectively [31]. Globally, IDC is the most prevalent histological type, accounting for up to 75% of cases, and ILC accounts for up to 15% of cases [39]. As for tumor size, 80% of the studied tumors were between two to five centimeters and 16% were less than 2 centimeters. This is in contrast with studies in developed countries, in which smaller tumor sizes (< 2 cm) were more common (58.4% and 42.6% respectively) [5, 40]. This denotes that low screening, delay in seeking medical advice and ignorance of BC symptoms among Egyptian females.

Lymphovascular invasion was noted in 57.3% of the studied BC patients and the most prevalent histologic grade in the studied BC patients was grade 2 (77.3%). This agrees with an Egyptian study *by Metwally et al.* in which showing 58.8% LVI while higher grade 2 prevalence (93.8%) [41]. 

Regarding tumor stage, 74.7% of the studied BC tumors showed early stage (28% were stage I and 46.7% were stage II) while 25.3% were late stage (stage III). No patients with grade IV were present in this study. Compared to a study by *Rostom and colleagues* conducted in Alexandria, our study had a higher percentage of early-stage BC (74.7% vs. 55.2%) and lower percentage of late-stage BC (25.3% vs. 37.6%) [42]. This could be attributed to the differences in patients cohort attending the radiological center which conducted the study. In *Azim et al.* metanalytic study of Egyptian females, he reported high rates of BC in patients with younger age and advanced stages (45%) [31]. 

Passing into the core of this study, which was to detect the prevalence of BLV, EBV and HPV in BC tissue of Egyptian patients, this study has demonstrated low prevalence of these three viruses in BC tissue as BLV, EBV and HPV were detected in (22.7, 14.7%, 6.7%) BC tissues respectively accounting for 37.3% of BC cases with no statistically significance difference found.

In this study, BLV retrotranscribed DNA was detected in 22.7% of BC tissues vs. 16% of BC free tissues. However, a lower prevalence was reported in other studies in Egypt (16% vs. 4%, *P* = 0.04) and Jordan (18.4% vs. 0%, *P* > 0.05) [43, 44]. A statistical association between BLV and BC was suggested by a recent metanalysis in the presence of diversity between the 17 studies [45]. Globally the detection rate of BLV in BC tissues varies from 0 to 60%. The variation of the prevalence of BLV could be due to the variation of the prevalence of contaminated dairy cattle with BLV and the consumption rate of dairy products between populations in different areas [46]. 

EBV DNA was detected in 14.7% of BC tissues vs. 8% of BC free tissues. A higher EBV prevalence were reported in Egyptian studies by *El-Naby et al.* (24%vs 14%) [47], and *Metwally et al.* (37% in BC tissues only) with no statistical relevance [41]. A recent systematic review of 24 studies has pointed out a statistical difference in EBV presence in BC vs. non-BC tissues (28% vs. 8%) with a wide geographical difference in prevalence (4.6–64%) [48]. 

In the current study, HPV DNA was detected in 6.7% of BC tissues only. A higher HPV prevalence was reported in Egypt by *El-Sheikh et al.* (22.2%) and *Metwally et al.* (41%) also in BC tissues only [41, 49]. A recent metanalysis of 23 studies revealed HPV prevalence to be 22% in BC tissues vs. 9% in nn-malignant tissues with statistical significance (*P* < 0.01) [50]. Another updated metanalysis reported 26% prevalene of HPV in BC with different heterogeneity between studies [51]. HPV detection rate in BC varies from 0 to 86% [29]. HPV16 was the detected genotype in two BC tissues in our study. HPV16 is the high-risk type most found in BC along HPV 18 and 33 [52, 53].

Regarding the association of BLV, EBV and HPV presence with different BC pathological parameters, Single viral detection was not found to be statistically significant in relation to clinicopathological parameters. These results run in parallel to several studies in Egypt and other countries. In Egypt, *Elmatbouly et al.* reported no significant association between BLV and tumor grade, stage, or LN metastasis, and *Metwally et al.*, found no association between EBV or HPV detection and pathological parameters [41, 43]. Also, *EL-Sheikh et al.* reported no significance between HPV positivity and histopathologic type, tumor size, grade or lymph node metastasis [49]. In Brazil, *Schwingel* et al. found no association between BLV DNA and tumor prognostic markers [54]. *Naushed et al.*, in Pakistan, found no association between HPV or EBV positivity as regards histologic type and hormone receptor expression [55]. *Glenn et al.* reported, no significance between EBV or HPV positivity and tumor grade or hormonal receptor expression [56]. 

Regarding viral co-detection, BLV and EBV were simultaneously found in 5.3% of the studied BC tissues. No research was found studying the coexistence of these two viruses and BC. BLV co-viral relationships research gap should be underscored.

EBV and HPV DNA were simultaneously found in one BC tissue (1.3%). Likewise, EBV and HPV coprevalence was found in 1.8% in a study in Jordan [44]. *Metwally et al.*, in Egypt, and *Naushed et al.*, in Pakistan, reported higher EBV- HPV copresence (10% and 9.2%) respectively [41, 55]. A higher codetection were reported by a study in Qatar and Lebanon (47% and 30%) respectively with significant association to tumor grade [57, 58]. 

Interestingly, it was noted that detection of a single virus is more common among the older age group (≥ 50 years), while multiple viral detection was found more among the younger age group (< 50 years) with a statistical significance (*P* = 0.026). These results are mainly in agreement with studies by *Metwally et al.* and *Glenn et al.* in which oncogenic viruses copresence was associated with a younger age [41, 56]. This association could be explained due to multiple observations. First, as previously mentioned, breast cancer has been reported to be diagnosed a decade earlier in Egypt than in Western countries with advanced stages [3, 4, 31]. Second, unique and high number of mutations were detected in BRCA1 and BRCA2 genes females in developing countries which might be related to high exposure to certain risk factors including viruses [3]. Third, EBV and HPV were previously detected at younger age in breast cancer [56, 59]. This could be attributed to increased behavioral exposure through early exposure to EBV by or continued HPV exposure in adulthood through sexual activity. Fourth, the 3 viral oncogenic potentials and BC development have been frequently suggested in literature and clarified above. Remarkably in our study, EBV detection was more found in BC patients with younger age as well as being found in all tumors with positive dual viral coexistence which was the reason behind the statistical significance relationship between multiple viral presence and young age. It is worth reporting that EBV has been detected in multiple epithelial were previously at a younger age. A meta-analysis detected a significant association between EBV detection in gastric carcinoma in younger patients and in Caucasians [60]. Also, a head and neck cancer study reported the coprevelance of EBV and HR HPV in younger age [61]. This may highlight that certain risk factors as viral exposure as well as certain biological and immunological individual determinants might reactivate this ubiquitous latent virus in adulthood rather than late reactivation by the elderly immune suppression state. No study has explored BLV viral copresence in breast cancer. We hope that this study will help in increasing the growing knowledge of this virus with suggested oncogenic potential.

## Conclusion

Low rates of BLV, EBV and HPV presence were detected in BC tissues. Nevertheless, LVI and stage III were more commonly seen among tissues with positive viral detection vs. those which were negative.

Scarce studies have investigated multiple viral associations with BC. Exploring this complex role and including the emerging less studied BLV, which was the highest to be detected both solely and as a co-virus, is unique to the current study in our country and worldwide. This study adds to growing knowledge of a possible synergetic role between BLV, the ubiquitous EBV; condemned in multiple epithelial and non-epithelial malignancies, and the oncogenic HR HPV especially in young age.

**limitations and recommendations**: Limitations of the study include the small size of specimens, due to lack of project funding, might be responsible for the low viral detection rates. Nevertheless, the low detection rates can also be attributed to the low viral copy number in the infected cell in comparison to the host genetic material. Also, the location in the breast might be another factor contributing to low detection HPV rate as it is extremely low in BC, 2000 fold less, than in cervical cancer. Also, lack of fresh breast tissues for examination might add to the low detection rates. However, housekeeping genes were detected in all included FFPE which denotes good DNA integrity. Prevalence of BLV among humans was not detected due to the retrospective nature of the study focusing on exploring an emerging less studied virus and its association with other ubiquitous oncogenic viruses in BC development.

Increasing awareness to BC screening in women above 40 years is an urgent priority. Strengthening HPV vaccine coverage and elimination of BLV from dairy products should be encouraged and longitudinal studies designed to detect changes in BC prevalence are recommended. Further multicenter research combining studies pooled results and analyzing viral proteins interactions to formulate possible oncogenic effects are also recommended to tailor targeted therapy.

## Electronic supplementary material

Below is the link to the electronic supplementary material.

Supplementary Material 1

Supplementary Material 2

## Data Availability

No datasets were generated or analysed during the current study.
