# Correction: Detection of bovine leukemia virus, Epstein-Barr virus and human papillomavirus in breast cancer tissues of Egyptian patients

**DOI:** 10.1186/s13027-025-00692-w

**Published:** 2025-08-26

**Authors:** May Raouf, Salwa Kamal, Rawan Elsayed, Inass Zaki, Dina Kholeif

**Affiliations:** 1 Medical Microbiology and Immunology Department, Faculty of Medicine, Alexandria University, 0 Khartoum square, Azarita Medical campus, Alexandria, 21131 Egypt; 2 Pathology Department, Faculty of Medicine, Alexandria University, Alexandria, Egypt

**Correction to**: ***Infectious Agents and Cancer*** **(2025) 20:43**

10.1186/s13027-025-00674-y

In this article a duplication of Fig. 2 appeared as Fig. 1 due to a typesetting mistake.

Incorrect Figure 1:

**Figure:**
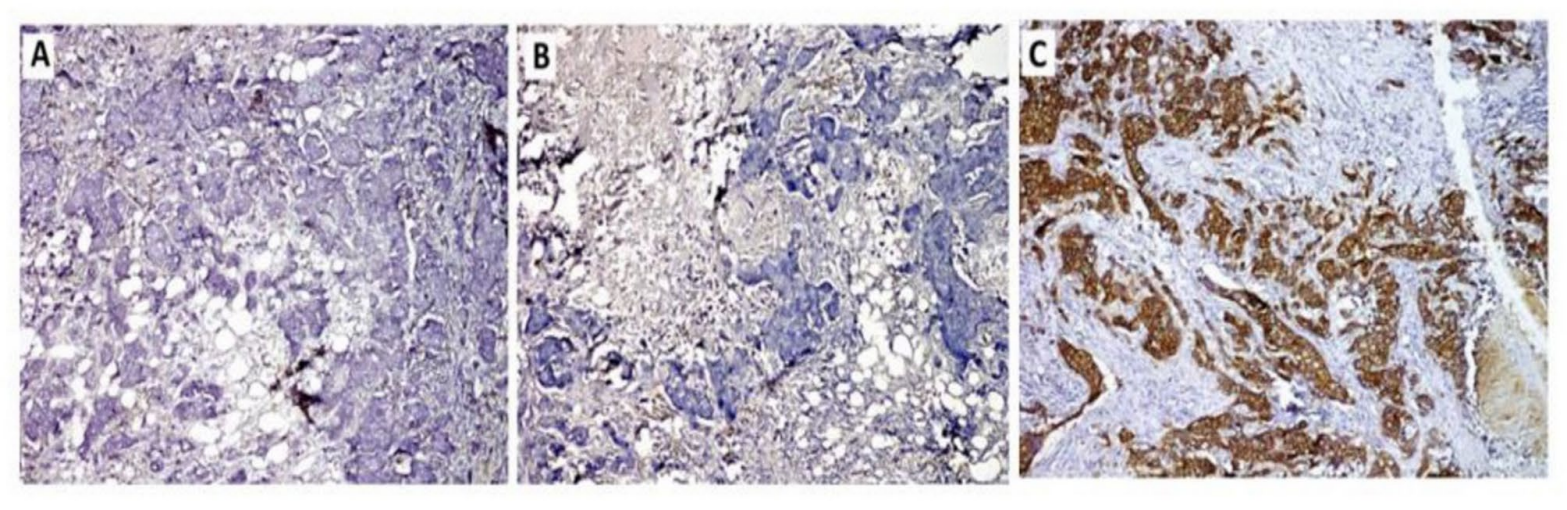

Correct Figure 1:

**Fig. 1:**
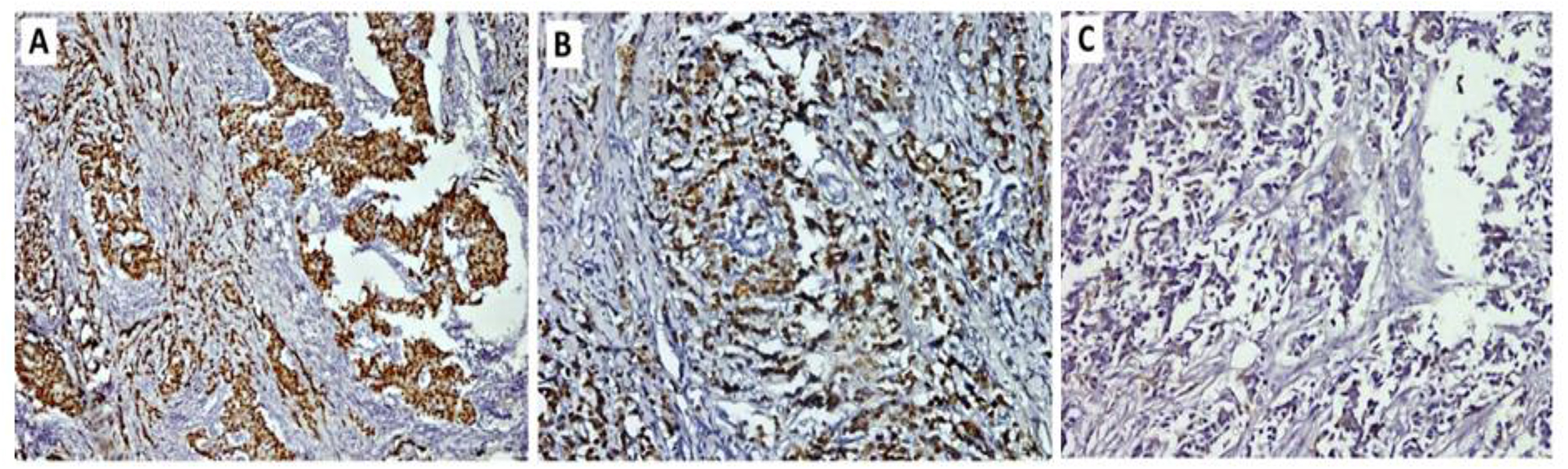
A case of invasive ductal carcinoma of no special type NST, G2 showing **A**: positive nuclear staining for ER antibody, **B**: positive nuclear staining for PR antibody, **C**: negative membranous staining (score 0) for Her2-neu antibody (Immunoperoxidase **A**: x100, **B**: x200, **C**: x200)

The original article has been corrected and the publisher apologises to the authors and readers for the inconvenience caused by the error.

