# Supplementary material for: Detection of bovine leukemia virus, Epstein-Barr virus and human papillomavirus in breast cancer tissues of Egyptian patients
Source: Infect Agent Cancer. 2025 Jul 1;20:43. doi: 10.1186/s13027-025-00674-y (PMC12218089; doi:10.1186/s13027-025-00674-y)

www.qiagen.com

Melt Report

Experiment Information

Run Name	DR ROWAN SALAH 2023-02-08 (1)
Run Start	2/8/2023 10:25:31 AM
Run Finish	2/8/2023 11:36:03 AM
Operator	
Notes	
Run On Software Version	Rotor-Gene Q Software 2.3.3.5 TECHNICIAN
Run Signature	The Run Signature is valid.
Gain Green	9.33
Machine Serial No.	0115301

Melt Information

Digital Filter	Light
Imported Analysis Settings	
Sample Page	Page 1
Temp. Threshold	80°C
Threshold	5.

Melt data for Melt A.Green

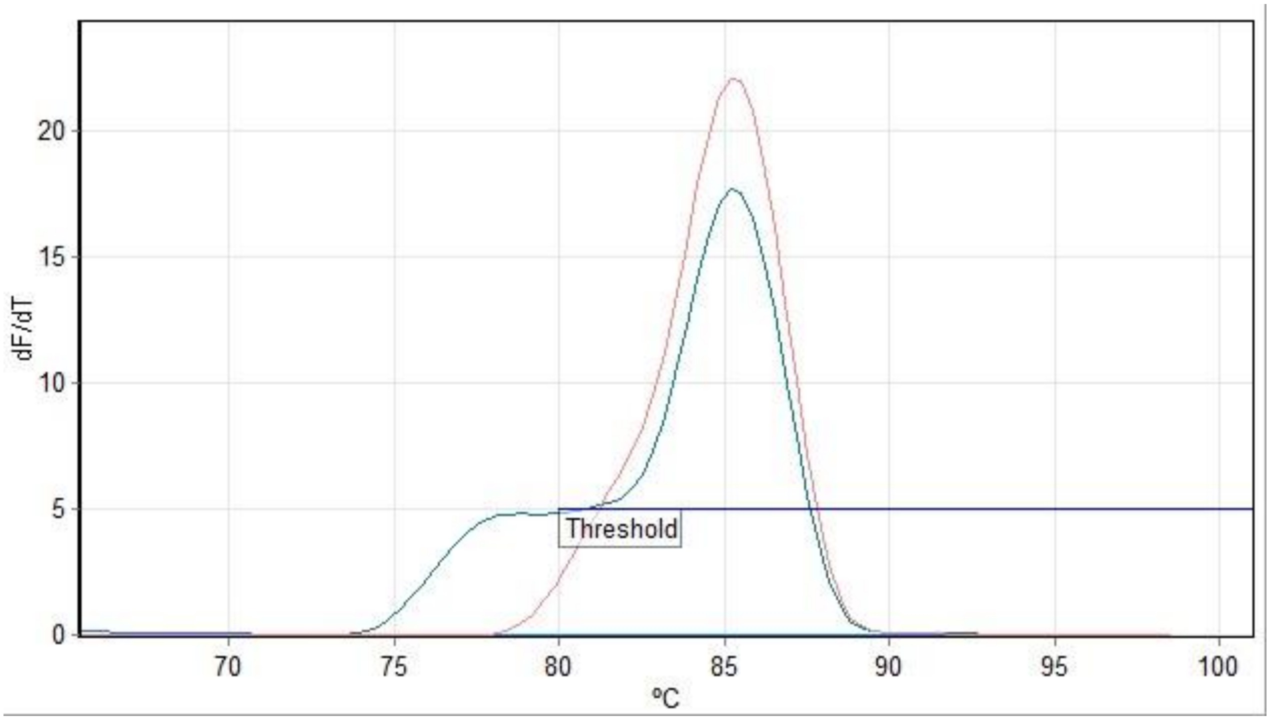

Color	Name	Peak 1
	Negative control	
	493 B globin	85.3
	1434 B globin	85.3

Bin Name	Temperature	Sample No.	Sample Name	Peak

This report was generated by Rotor-Gene Q Series Software 2.3.3 (Build 5)
Copyright ©2013 QIAGEN GmbH. All Rights Reserved.

www.qiagen.com

Melt Report

Experiment Information

Run Name	DR ROWAN SALAH 2023-02-09 (1)
Run Start	2/9/2023 9:17:38 AM
Run Finish	2/9/2023 10:27:52 AM
Operator	
Notes	
Run On Software Version	Rotor-Gene Q Software 2.3.3.5 TECHNICIAN
Run Signature	The Run Signature is valid.
Gain Green	10.
Machine Serial No.	0115301

Melt Information

Digital Filter	Light
Imported Analysis Settings	
Sample Page	Page 1
Temp. Threshold	80°C
Threshold	8.78681

Melt data for Melt A.Green

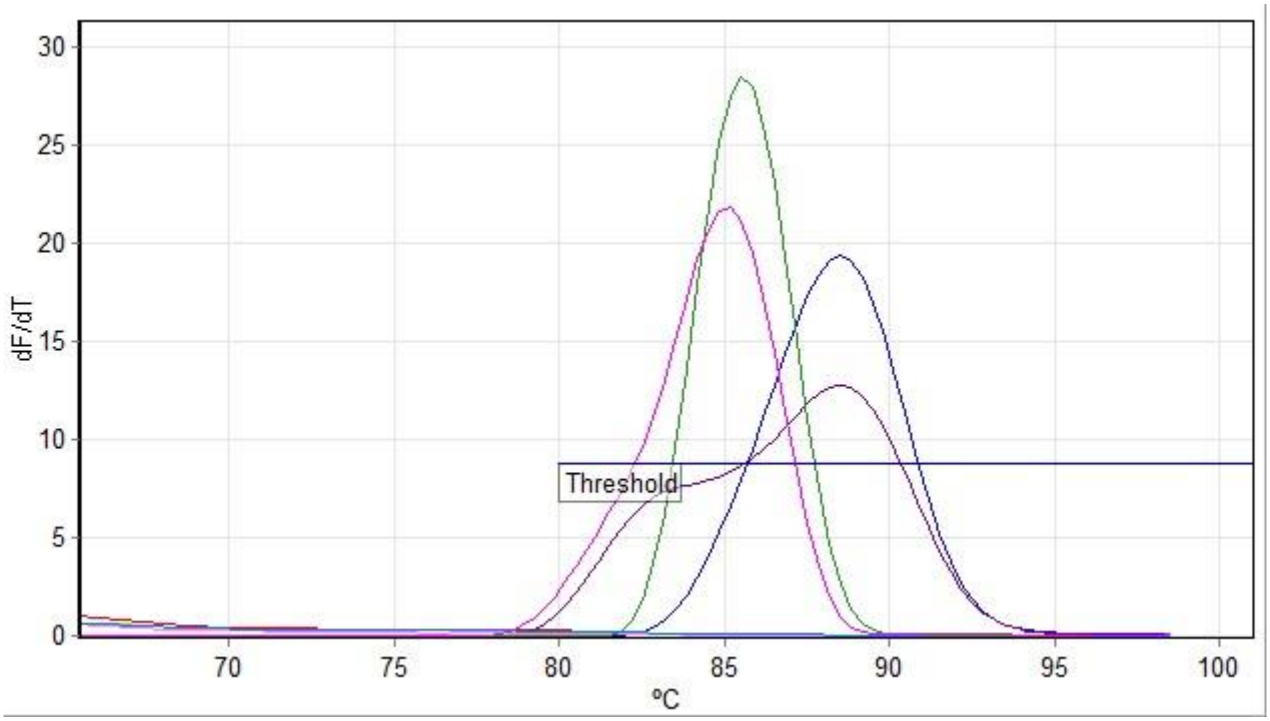

Color	Name	Peak 1
	1435 BLV	
	1527 BLV	
	1786 BLV	88.5
	1797 BLV	88.5
	Negative control	
	1537 BLV	
	Internal control	85.5
	Internal control	85.0

Bin Name	Temperature	Sample No.	Sample Name	Peak

This report was generated by Rotor-Gene Q Series Software 2.3.3 (Build 5)
Copyright ©2013 QIAGEN GmbH. All Rights Reserved.

www.qiagen.com

Melt Report

Experiment Information

Run Name	DR ROWAN SALAH 2023-10-11 (1)
Run Start	10/11/2023 6:09:59 AM
Run Finish	10/11/2023 7:19:12 AM
Operator	
Notes	
Run On Software Version	Rotor-Gene Q Software 2.3.3.5 TECHNICIAN
Run Signature	The Run Signature is valid.
Gain Green	6.67
Machine Serial No.	0115301

Melt Information

Digital Filter	Light
Imported Analysis Settings	
Sample Page	Page 1
Temp. Threshold	80°C
Threshold	0.43917

Melt data for Melt A.Green

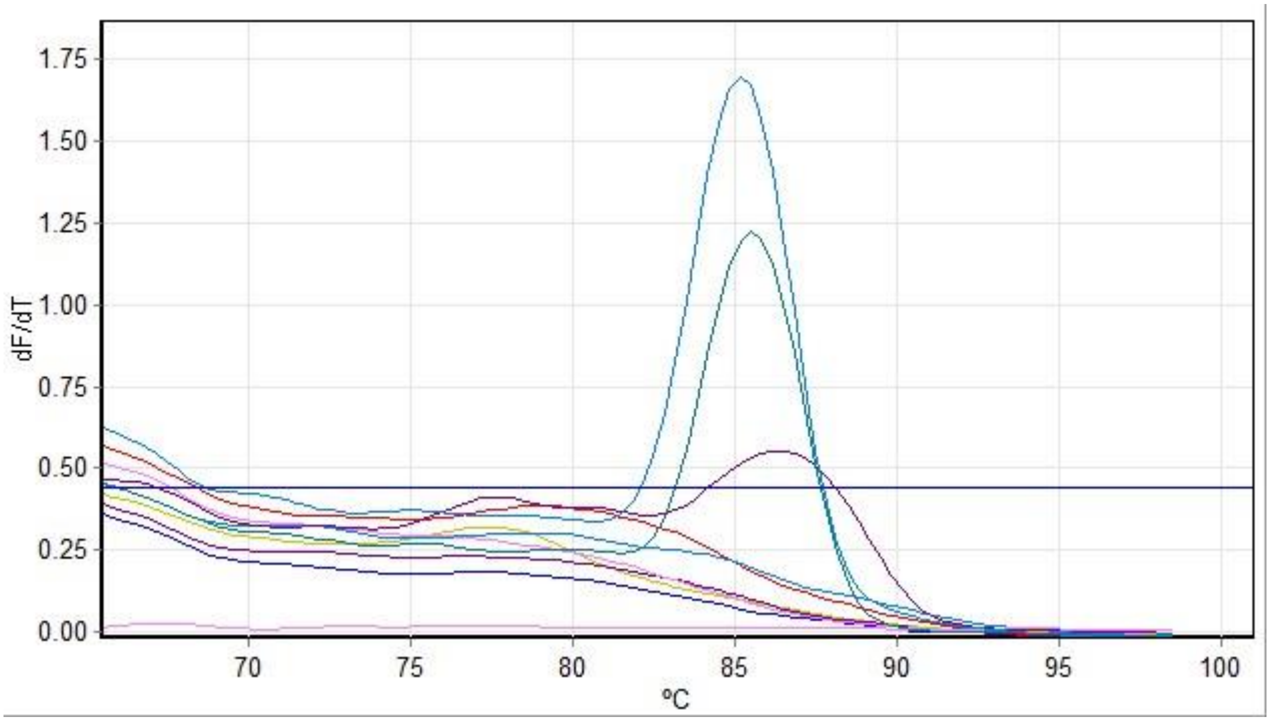

Color	Name	Peak 1
	8 EBV	
	9 EBV	
	10 EBV	
	11 EBV	
	12 EBV	
	13 EBV	
	16 EBV	86.5
	Negative control	
	Internal control	85.2
	Internal control	85.5

Bin Name	Temperature	Sample No.	Sample Name	Peak

This report was generated by Rotor-Gene Q Series Software 2.3.3 (Build 5)
Copyright ©2013 QIAGEN GmbH. All Rights Reserved.

www.qiagen.com

Melt Report

Experiment Information

Run Name	DR ROWAN SALAH HPV 45 2023-11-12 (1)
Run Start	11/12/2023 9:57:09 AM
Run Finish	11/12/2023 11:16:40 AM
Operator	
Notes	
Run On Software Version	Rotor-Gene Q Software 2.3.3.5 TECHNICIAN
Run Signature	The Run Signature is valid.
Gain Green	8.
Machine Serial No.	0115301

Melt Information

Digital Filter	Light
Imported Analysis Settings	
Sample Page	Page 1
Temp. Threshold	70°C
Threshold	3.06957

Melt data for Melt A.Green

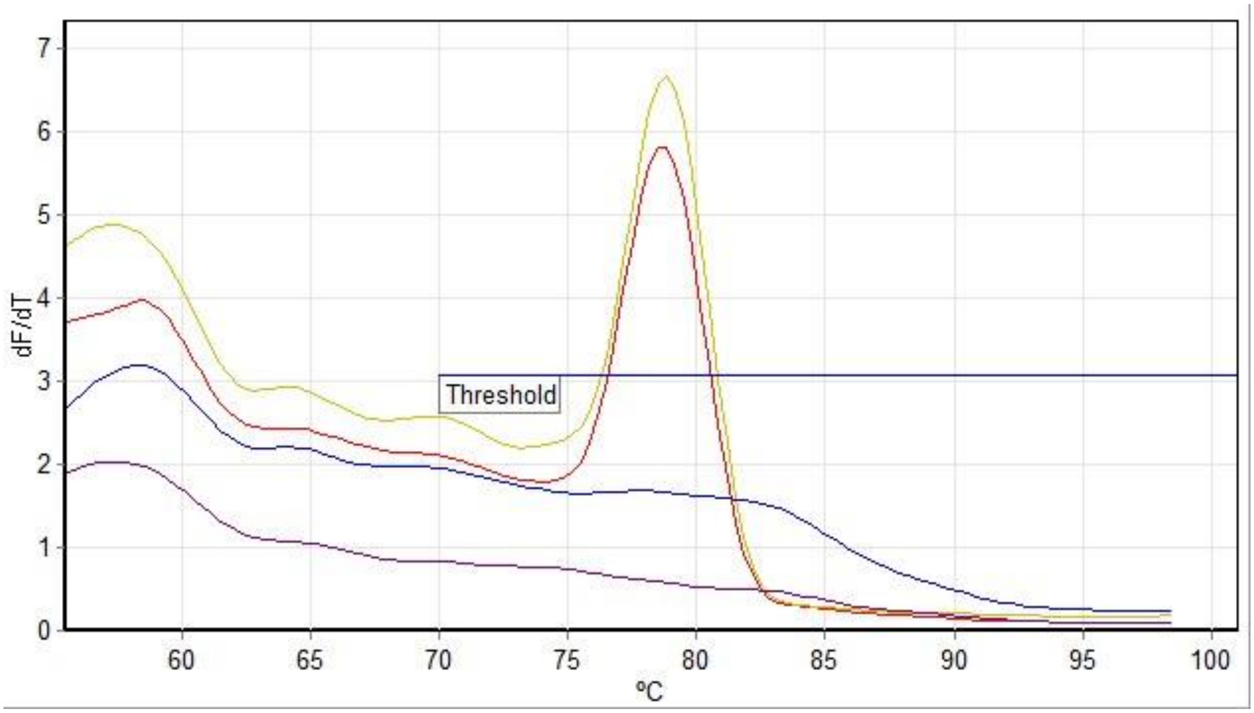

Color	Name	Peak 1
	22 HPV	78.7
	13 HPV	78.8
	8 HPV	
	Negative control	

Bin Name	Temperature	Sample No.	Sample Name	Peak

This report was generated by Rotor-Gene Q Series Software 2.3.3 (Build 5)
Copyright ©2013 QIAGEN GmbH. All Rights Reserved.

Allplex™ HPV28 Detection

**Sample No**	1		
**Patient Id**	faculty of medicine	**Patient Name**	------
**Well**	[A01]	**Data Analysis Time**	2025-04-01

- SAMPLE -

...	FAM						HEX						Cal Red 610						Quasar 670				Quasar 705						Quas...	
-	66	C(t)	45	C(t)	58	C(t)	51	C(t)	59	C(t)	16	C(t)	33	C(t)	39	C(t)	52	C(t)	35	C(t)	18	C(t)	56	C(t)	68	C(t)	31	C(t)	IC	C(t)
	-	N/A	-	N/A	-	N/A	-	N/A	-	N/A	-	N/A	-	N/A	-	N/A	-	N/A	-	N/A	-	N/A	-	N/A	-	N/A	-	N/A	+	25.7

- POSITIVE CONTROL -

- NEGATIVE CONTROL -

- GRAPH -

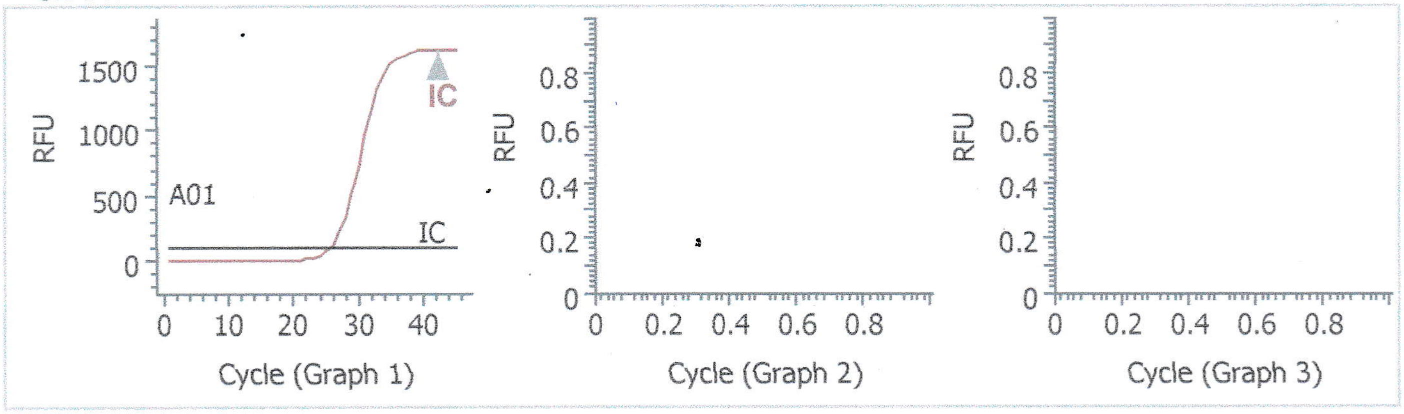

Allplex™ HPV28 Detection

**Sample No**	1		
**Patient Id**	faculty of medicine	**Patient Name**	--------
**Well**	[A02]	**Data Analysis Time**	2025-04-01

- SAMPLE -

...	FAM						HEX						Cal Red 610						Quasar 670				Quasar 705						Quas...	
-	26	C(t)	69	C(t)	73	C(t)	42	C(t)	82	C(t)	53	C(t)	43	C(t)	54	C(t)	70	C(t)	61	C(t)	6	C(t)	44	C(t)	40	C(t)	11	C(t)	IC	C(t)
	-	N/A	-	N/A	-	N/A	-	N/A	-	N/A	-	N/A	-	N/A	-	N/A	-	N/A	-	N/A	-	N/A	-	N/A	-	N/A	-	N/A	+	26.[illegible]

- POSITIVE CONTROL -

- NEGATIVE CONTROL -

- GRAPH -

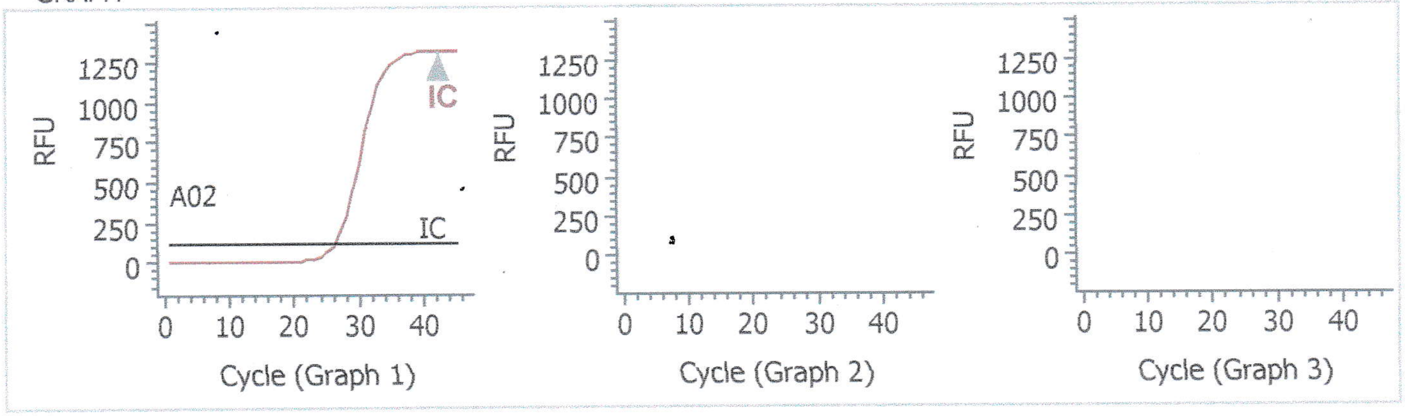

Anyplex™ II HPV28 Detection (8 strip)

Sample No			
Patient Id		Patient Name	2
Well	[C01, C07]	Data Analysis Time	2024-11-23

- SAMPLE -

Auto Interpretation	FAM			HEX			Cal Red 610			Quasar 670		Quasar 705			
16	66	45	58	51	59	16	33	39	52	35	18	56	68	31	IC
	-	-	-	-	-	++	-	-	-	-	-	-	-	-	++
	26	69	73	42	82	53	43	54	70	61	6	44	40	11	IC
	-	-	-	-	-	-	-	-	-	-	-	-	-	-	++

· High Risk : 16

- POSITIVE CONTROL -

- NEGATIVE CONTROL -

- GRAPH -

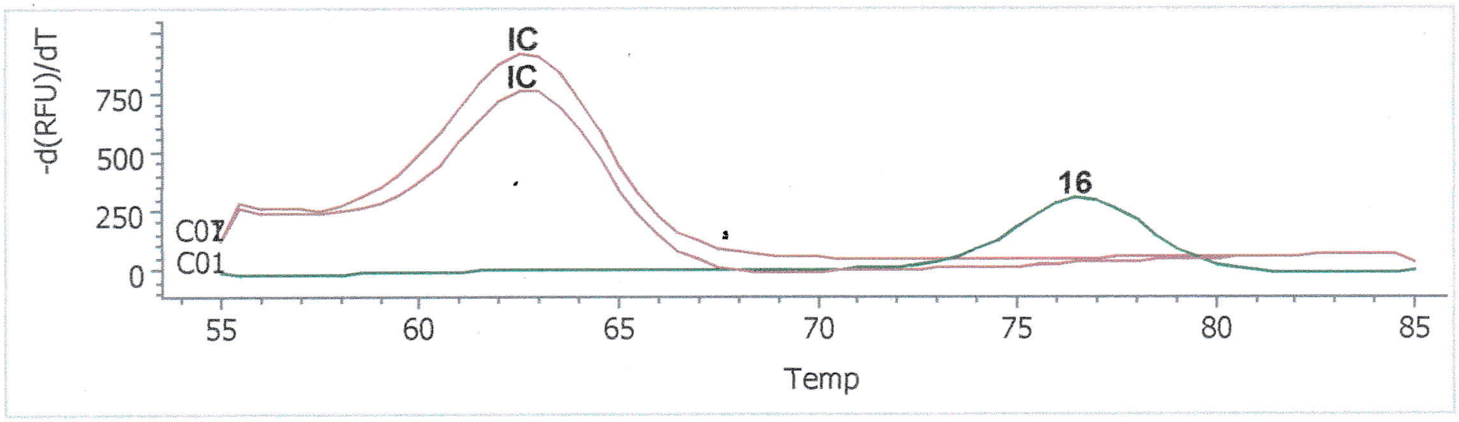

Supplement: Supplementary file 1 — Supplementary Material 1 [file 13027_2025_674_MOESM1_ESM.pdf]
