# Supplementary material for: Detection of bovine leukemia virus, Epstein-Barr virus and human papillomavirus in breast cancer tissues of Egyptian patients
Source: Infect Agent Cancer. 2025 Jul 1;20:43. doi: 10.1186/s13027-025-00674-y (PMC12218089; doi:10.1186/s13027-025-00674-y)
Supplement: Supplementary file 2 — Supplementary Material 2 [file 13027_2025_674_MOESM2_ESM.pdf]

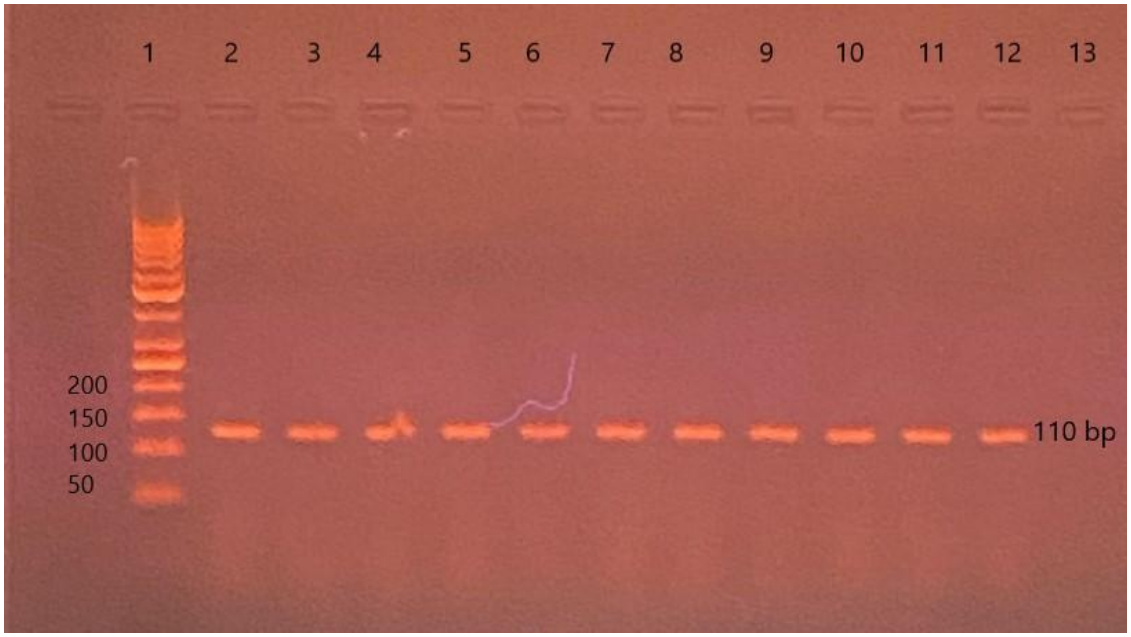

Figure (1): Human housekeeping β-globin gene positive PCR products bands (110-bp) on 2% agarose gel.

Well 1: 50bp DNA ladder. Wells 2-11: positive samples.

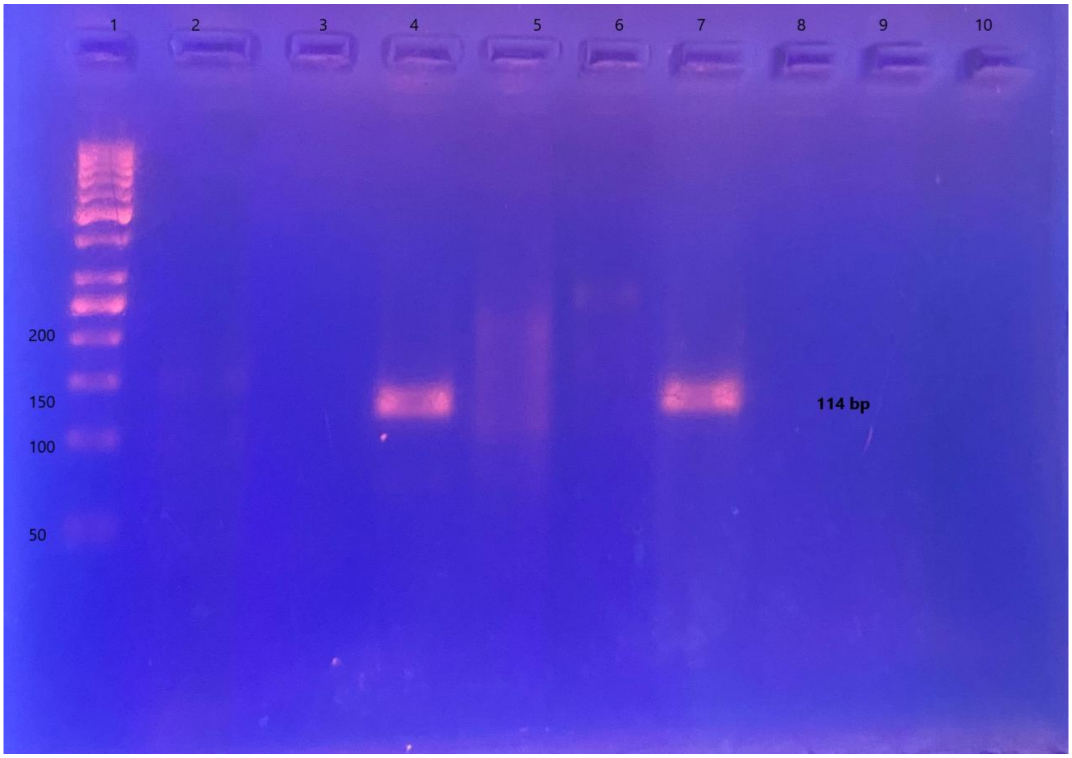

Figure (2): BLV positive PCR products bands (114-bp) on 2% agarose gel. Well 1: 50bp gel ladder. Wells 4 & 7: BLV positive samples.

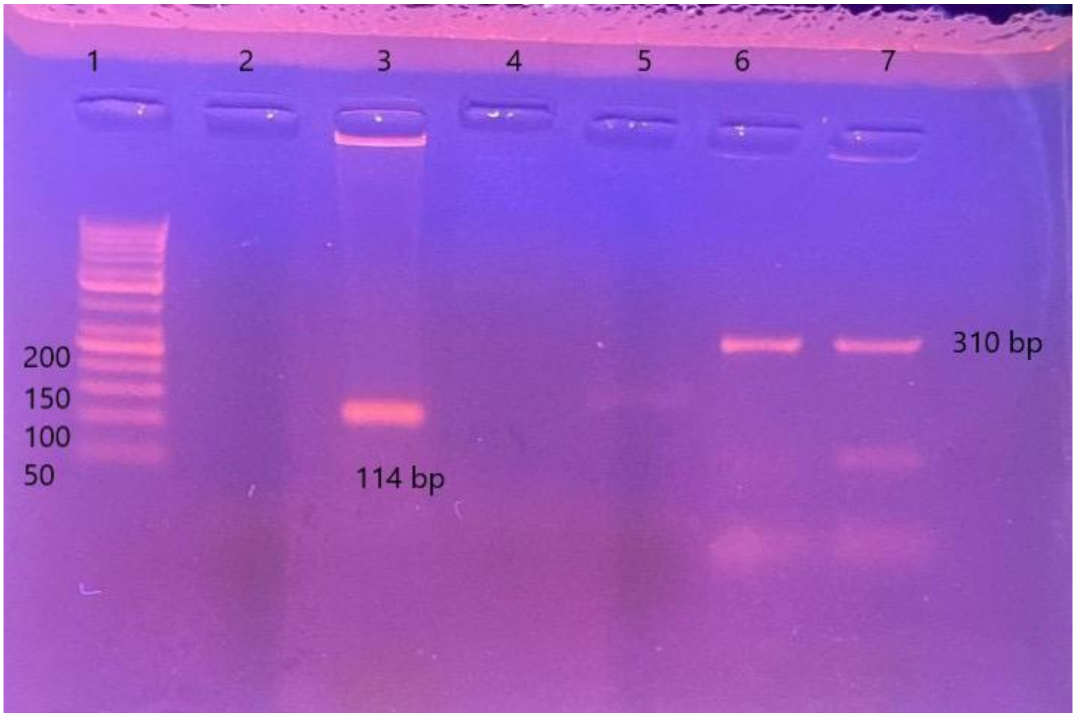

Figure (3): BLV and EBV positive PCR products bands (114 & 310bp) on 2% agarose gel. Well 1: 50bp gel ladder. Well 3: BLV positive sample. Wells 6 & 7: EBV positive samples.

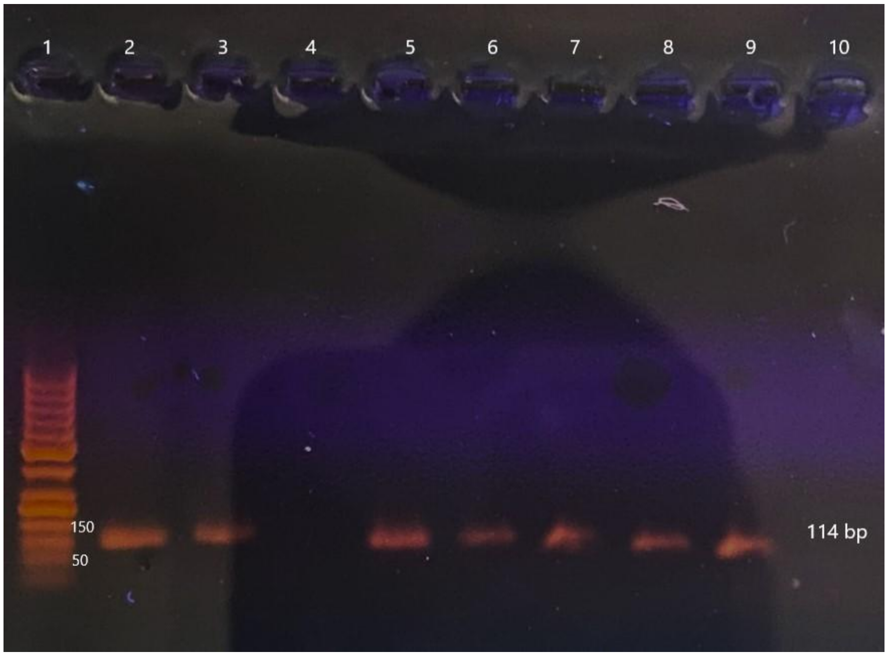

Figure (4): BLV positive PCR products bands (114 bp PCR product) on 2% agarose gel. Well 1: 50bp gel ladder. Wells 2, 3, 5-10: BLV positive samples.

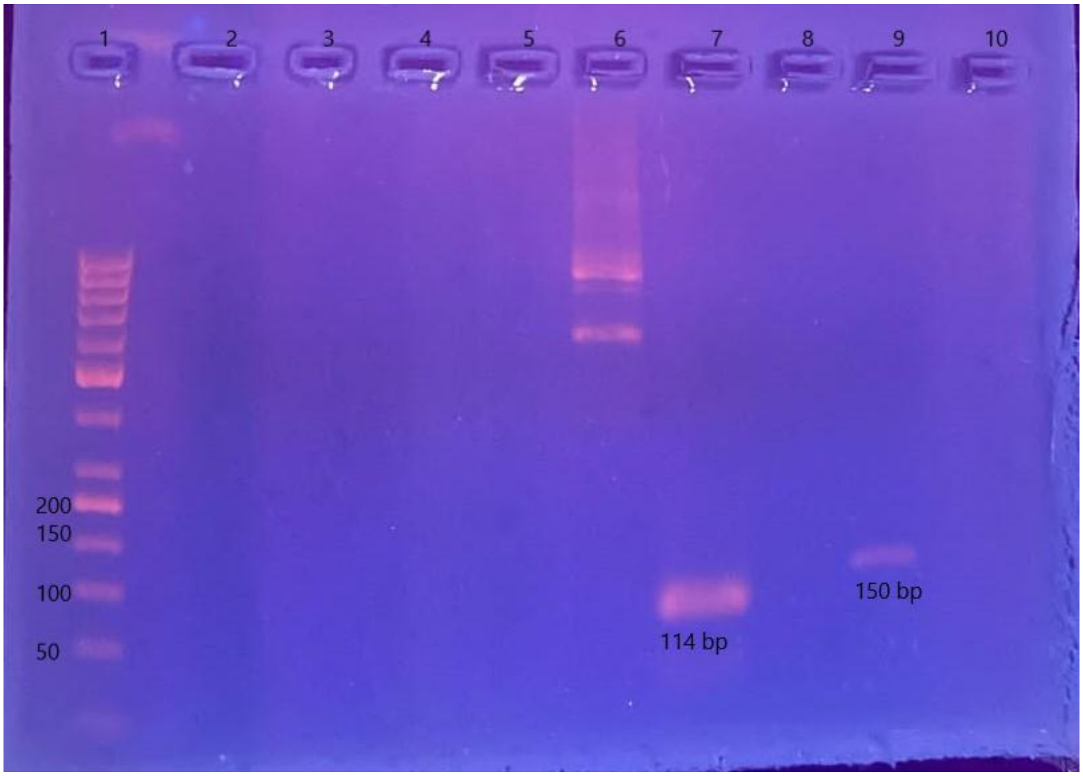

Figure (5): BLV and HPV positive PCR products bands (114 and 150 bp) on 2% agarose gel. Well 1: 50bp gel ladder. Well 7: BLV positive sample. Well 9: HPV positive sample.
